# Supplementary material for: Human Placenta Exosomes: Biogenesis, Isolation, Composition, and Prospects for Use in Diagnostics
Source: Int J Mol Sci. 2021 Feb 22;22(4):2158. doi: 10.3390/ijms22042158 (PMC7926398; doi:10.3390/ijms22042158)
Supplement: Supplementary file 1 [file ijms-22-02158-s001.pdf]

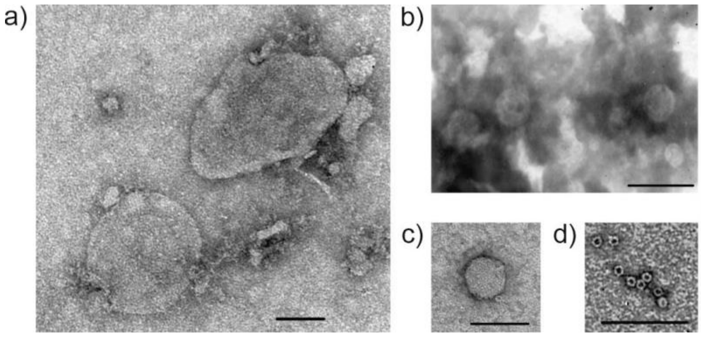


**Figure S1.** Structural components of the exosomes preparations after differential centrifugation. Transmission electron microscopy, negative staining. (**a)** Large membrane structures having a diameter of more than 100 nm; (**b**) aggregated proteins and small vesicles; (**c**) "non-vesicles"; (**d**) ferritin. The length of the scale bar is corresponding to 100 nm.

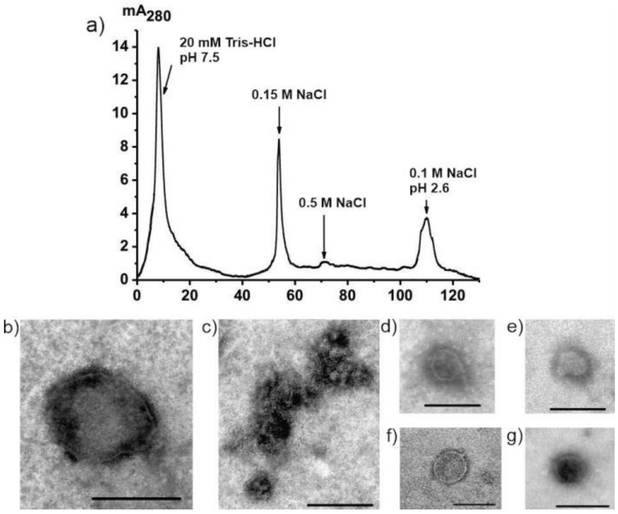


**Figure S2.** (**a**) Affinity chromatography of the exosome preparations on anti-CD81-Sepharose, elution conditions are indicated in the picture, (—) absorbance at 280 nm ($mA_{280}$). (**b–g)** Structural components of exosomes preparations after affinity chromatography: fraction eluted with 20 mM Tris-HCl pH 7.5 contains large vesicles (**b**) and aggregated proteins (**c**); fraction eluted with 0.15 M NaCl contains exosomes (**d, f**); fraction eluted with 0.1 M Gly-HCl pH 2.6 contains small amount exosomes (**g**) and "non-vesicles" (**e**). Transmission electron microscopy, negative staining. The length of the scale bar scale corresponds to 200 nm (**b**) and 100 nm (**c–g**).
